# Transgenerational memory of gene expression changes induced by heavy metal stress in rice (*Oryza sativa* L.)

**DOI:** 10.1186/s12870-019-1887-7

**Published:** 2019-06-27

**Authors:** Weixuan Cong, Yiling Miao, Lei Xu, Yunhong Zhang, Chunlei Yuan, Junmeng Wang, Tingting Zhuang, Xiuyun Lin, Lili Jiang, Ningning Wang, Jian Ma, Karen A. Sanguinet, Bao Liu, Sachin Rustgi, Xiufang Ou

**Affiliations:** 1 Key Laboratory of Molecular Epigenetics of MOE and Institute of Genetics & Cytology, Northeast Normal University, Changchun, 130024 China; 2 Jilin Academy of Agricultural Sciences, Changchun, 130033 China; 3 Jilin Agriculture University, Changchun, 130000 China; 4 Department of Crop and Soil Sciences, Washington State University, Pullman, WA 99164 USA; 5 Department of Plant and Environmental Sciences, Clemson University, Pee Dee Research and Education Center, Florence, SC 29506 USA

**Keywords:** Heavy metal stress, Transgenerational memory, Gene expression, DNA methylation

## Abstract

**Background:**

Heavy metal toxicity has become a major threat to sustainable crop production worldwide. Thus, considerable interest has been placed on deciphering the mechanisms that allow plants to combat heavy metal stress. Strategies to deal with heavy metals are largely focused on detoxification, transport and/or sequestration. The P_1B_ subfamily of the Heavy Metal-transporting P-type ATPases (HMAs) was shown to play a crucial role in the uptake and translocation of heavy metals in plants. Here, we report the locus-specific expression changes in the rice *HMA* genes together with several low-copy cellular genes and transposable elements upon the heavy metal treatment and monitored the transgenerational inheritance of the altered expression states. We reveal that plants cope with heavy metal stress by making heritable changes in gene expression and further determined gene-specific responses to heavy metal stress.

**Results:**

We found most *HMA* genes were upregulated in response to heavy metal stress, and furthermore found evidence of transgenerational memory via changes in gene regulation even after the removal of heavy metals. To explore whether DNA methylation was also altered in response to the heavy metal stress, we selected a *Tos17* retrotransposon for bisulfite sequencing and studied its methylation state across three generations. We found the DNA methylation state of *Tos17* was altered in response to the heavy metal stress and showed transgenerational inheritance.

**Conclusions:**

Collectively, the present study elucidates heritable changes in gene expression and DNA methylation in rice upon exposure to heavy metal stress and discusses implications of this knowledge in breeding for heavy metal tolerant crops.

**Electronic supplementary material:**

The online version of this article (10.1186/s12870-019-1887-7) contains supplementary material, which is available to authorized users.

## Background

Plants are sessile organisms and are often confronted with a variety of stress factors simultaneously, which can dramatically decrease their yield and quality. In the recent years, heavy metal pollution, i.e., contamination of the natural environment with cadmium (Cd), chromium (Cr), copper (Cu), mercury (Hg), and zinc (Zn) has become a global problem, affecting about 235 million hectares of the arable land worldwide [1]. Heavy metals compromise crop productivity and pose a threat to human health via heavy metal accumulation in the food chain [2]. In plants, heavy metals interfere with several metabolic processes including photosynthesis, water relations, and nutrient uptake, resulting in reduced plant growth, stunting, and in some instances, death [3, 4]. Cu is an essential micronutrient; however, if present in excess it also causes toxicity to plants [5]. Cr is also a common metal contaminant in the Earth’s crust. While naturally occurring, Cr does not cause toxicity to plants, but excessive amounts can cause injury [6]. Cd and Hg are both non-essential and toxic elements for plant growth and human health. These elements are almost ubiquitously present at low levels in the environment but have now started to accumulate due to anthropogenic activities. In its 25-year plan for the comprehensive prevention and control of heavy metals the Ministry of Environmental Protection of the People’s Republic of China listed Cd, Pb, Hg, and Cr as the major environmental pollutants, and pledged efforts to control their release to the environment (www.cleanairchina.org/file/loadFile/9.html). Parallelly, in view of the public health concern, in the report on the National Food Safety Standard Limits on contaminants in food (GB 2762–2017) the National Standards of the People’s Republic of China, made recommendations on the maximum tolerable amount of Cu (10 mg kg^− 1^), Cr (1.0 mg kg^− 1^), Cd (0.2 mg kg^− 1^), and Hg (0.02 mg kg^− 1^) in rice grains.

Since heavy metal toxicity has become one of the major challenges in increasing crop productivity, investigating heavy metal tolerance genes and stacking them in a single genetic background, have become a major theme of plant breeding research. Over the course of evolution, plants have developed different strategies to overcome heavy metal toxicity. For example, relatively low levels of metals are present in shoots by either restricting translocation of toxic metals, sequestration to vacuoles, or detoxification [7–12]. Conversely, some plants have developed exceptional abilities to translocate and accumulate heavy metals in their aboveground organs [13].

Recent research has revealed that the P_1B_ subfamily of Heavy Metal-transporting P-type ATPases (HMAs) play a crucial role in the uptake and translocation of heavy metals in plants [14, 15]. There are eight and nine members of P_1B_-ATPases in *Arabidopsis thaliana* and rice (*Oryza sativa* L.), respectively [16, 17]. Based on the metal-substrate specificity these ATPases can be divided into two subgroups: a zinc (Zn)/cobalt (Co)/cadmium (Cd)/lead (Pb) group and a copper (Cu)/silver (Ag) group [18]. In *A. thaliana* and rice, AtHMA1-AtHMA4 and OsHMA1-OsHMA3 belong to the former group whereas AtHMA5-AtHMA8 and OsHMA4-OsHMA9 belong to the latter group [18]. All members of the HMA family in *A. thaliana* have been functionally well characterized. The HMA family members exhibit differences in expression sub-cellular localization, and metal specificity and regulation, which all indicate unique functions within the gene family. For instance, AtHMA1, AtHMA5-AtHMA8 were reported to play a role in Cu homeostasis [19–22]. AtHMA2-AtHMA4 were involved in Cd translocation and sequestration [23–25]. In contrast, the rice HMA transporter family is not as well characterized. For instance, OsHMA1 and OsHMA9 were postulated to play a role in Zn transport [26, 27]. OsHMA2 and OsHMA3 were reported to be involved in the transportation of Cd [28–30], OsHMA4 and OsHMA5 have a function in Cu transport, loading, and detoxification [31, 32]. However, little research has been performed on OsHMA6, OsHMA7, and OsHMA8.

Modulation of gene expression is one rapid strategy to respond to environmental stresses. It has been repeatedly shown that heavy metal stress induces changes in gene expression. For instance, transcript profiling of the Cd-tolerant cultivar of Chinese flowing cabbage revealed numerous changes in gene expression in response to Cd treatment including upregulation of *HMA3* and *HMA4* [33]. Research in *Sedum plumbizincicola* showed elevated expression of the *SpHMA3* gene in response to Cd stress suggesting a role in Cd detoxification and normal growth of young leaves under Cd stress [34]. Similarly, in *Lycopersicum esculentum,* heavy metal transporters *COPT1* and *COPT2* could be induced to express under Cu stress [35]. Functional genomics tools have been extensively used to examine mechanisms conferring tolerance to various heavy metal stresses. In a recent report, genome-wide transcriptome analysis in rice showed dose-dependent changes in expression of metal ion transporter genes in response to Cd stress [36].

One way to maintain changes in gene expression is via epigenetic modification. Indeed, epigenetic variation contributes to phenotypic plasticity in response to the environmental changes [37]. In particular, DNA methylation is an important epigenetic marker, which regulates gene expression as an adaptive mechanism for survival under stress. In a recent study, genome-wide single-base resolution maps of methylated cytosines and transcript profile of Cd-treated rice was reported [38]. The study showed that most of the epigenetically regulated genes were transcriptionally activated under Cd stress, and many of these genes represent formerly characterized stress responders, metal transporters and transcription factors [38]. Despite initial progress, implementation of these epigenetic markers in plant breeding has stalled because the heritability of these makers has not yet been tested [37].

Since rice (*O. sativa* L.) is one of the major staple grains worldwide, increasing its productivity and nutritional quality is one of the foremost priorities. In the interest of ensuring food security and better nutritional quality, it is important to reduce the accumulation of toxic elements in rice grains [39, 40]. A deep understanding of the genes responsible for the sequestration of toxic elements can enable the development of crop varieties with reduced content of these elements in the edible plant parts. Our previously, work has shown that heavy metal stress (Cd, Cr, Cu, and Hg) could inhibit further shoot and root development of the ten-day-old rice seedlings and induce transgenerational changes in their DNA methylation pattern at specific loci [41]. Rice plants were treated with two different concentrations of Cd, Cr, Cu, or Hg to determine dose-dependent responses to these heavy metals. As expected, more hypomethylations were observed at specific-loci on the higher doses of Cd, Cr, and Cu but no change in DNA methylation pattern was witnessed upon Hg treatment. Remarkably, the progeny of the stressed plants exhibited enhanced tolerance to the same stress their progenitors experienced and showed the transgenerational inheritance of changes in the DNA methylation patterns [41]. The aim of this study was to address whether locus-specific changes in gene expression also take place in response to the heavy metal stress and whether different classes of genes have common or specific responses to heavy metal stress.

## Results

### Heavy metal stress induced locus-specific gene expression changes in the S_0_ plants

**Fig. 1:**
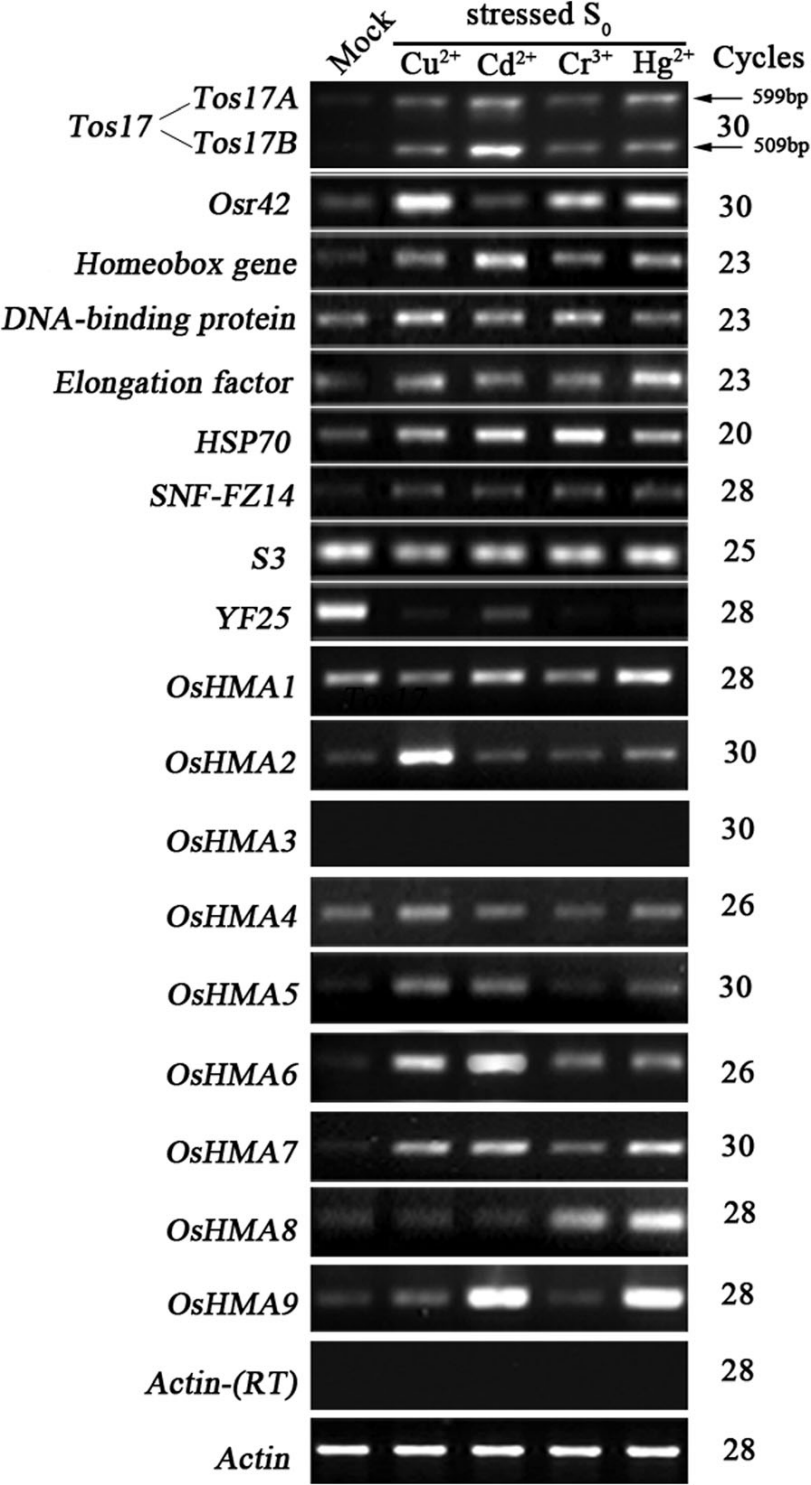
Alteration in the steady-state transcript abundance determined by semi-quantitative RT-PCR analysis in a set of 18 randomly selected genes, which include two transposable element genes (*Tos17* and *Osr42*), seven cellular genes (*homeobox gene*, *DNA-binding protein*, *Elongation factor*, *HSP70*, S*NF-FZ14*, *S3*, and *YF25*), and nine rice Heavy Metal-transporting P-type ATPases (*OsHMA1*-*OsHMA9*). The results were highly reproducible among the three independent RNA batches, and hence, only one was presented. Gene names are listed to the left and amplification cycles are labeled to the right of the gel. The rice *Actin* gene (Genbank accession # X79378) was used as a control for normalization of RNA input. Lack of genomic DNA was validated by the *Actin* gene on the template without RT

We previously showed that heavy metals elicit epigenetic changes in DNA methylation patterns of specific loci and in a transgenerational manner [41]. In the present study, we addressed whether locus-specific changes in gene expression also take place in response to the heavy metal stress and whether different classes of genes have common or specific responses to the heavy metal stress. To test this possibility, we assessed the expression of 18 randomly-distributed and functionally diverse genes by reverse transcription (RT)-PCR in the heavy-metal stressed rice seedlings (Fig. 1). Out of these 18 genes, two (*Tos17* and *Osr42*) were formerly tested by us to respond epigenetically to the heavy metal stress, seven (*Homeobox gene*, *DNA-binding protein*, *Elongation factor*, *HSP70*, S*NF-FZ14*, *S3*, and *YF25*) were randomly distributed cellular genes, and nine genes (*OsHMA1*-*OsHMA9*) were known to be heavy metal transporters. This panel of genes allows testing if global or specific transcriptional changes are involved in heavy-metal stress avoidance or mitigation in rice. In the S_0_ generation, plants for expression analysis were selected on the basis of the gel-blot analysis. Specifically, S_0_ plants that showed the most conspicuous modifications in DNA methylation patterns under Cu^2+^ (1000 μM), Cd^2+^ (1000 μM), Cr^3+^ (1000 μM) and Hg^2+^ (50 μM) treatments were selected for the expression analysis [41].

Interestingly, we found two rice TE (transposable element) genes, the *Tos17* and *Osr42* that showed significantly up-regulated expression under all or three of the four heavy metal treatments (Fig. 1 and Table 1). Specifically, for *Tos17*, there are two copies in wild-type rice cv. Nipponbare, one located on chromosome 10 dubbed *Tos17A*, and the other located on chromosome 7 called *Tos17B*. The two *Tos17* copies are identical except for a 90 bp insertion in *Tos17A* [42]. We designed gene-specific primers to study expression changes in the two copies under heavy metal stress. The results showed that the two copies of *Tos17* seldom exhibit activation of gene expression under all four (100%) heavy-metal treatments (S_0_ plants), particularly under Cd stress. Similarly, *Osr42* showed a significantly up-regulated expression under three (Cu, Cr, and Hg) of the four (75%) heavy metal treatments. The two TE genes exhibited contrasting expression patterns in Cd-treated plants, while *Tos17* showed the most conspicuous activation of gene expression, *Osr42* exhibited no change in expression.

**Table 1:** Gene expression changes observed for the 18 functionally diverse random genes in heavy metal treated seedlings of rice cv. Matsumae (S_0_ generation)

Gene name	Genbank acc.^a^	Chr.^a^	Gene expression changes observed in heavy metal treated plants of S_0_ generation^b^				
			Cu^2+^(1000 μm . L^−1^)	Cd^2+^ (1000 μm . L^−1^)	Cr^3+^ (1000 μm . L^− 1^)	Hg^2+^ (50 μm . L^− 1^)	Freq. (%)^c^
Transposable elements (TEs)							
*Tos17*	AC087545^(*Tos17A*)^	10	U	U	U	U	100
	AP008213^(*Tos17B*)^	7	U	U	U	U	100
*Osr42*	AF458768	4	U	N	U	U	75
Low copy protein-coding genes							
*Homeobox gene*	AB007627	2	U	U	U	U	100
*DNA-binding protein*	X88798	5	U	U	U	U	100
*Elongation factor*	D12821	7	U	U	U	U	100
*HSP70*	X67711	11	U	U	U	U	100
*SNF-FZ14*	DQ239432	7	U	U	U	U	100
*S3*	AY328087	12	N	N	N	N	0
*YF25*	DQ239435	11	D	D	D	D	100
Rice P_1B_ subfamily of Heavy Metal-transporting P-type ATPases (HMAs)							
*OsHMA1*	AP003935	6	N	U	N	U	50
*OsHMA2*	AP004278	6	U	N	N	N	25
*OsHMA3*	AP005246	7	–	–	–	–	–
*OsHMA4*	AP004184	2	N	N	N	N	0
*OsHMA5*	AL606647	4	U	U	N	U	75
*OsHMA6*	AP004836	2	U	U	U	U	100
*OsHMA7*	AP004376	8	U	U	U	U	100
*OsHMA8*	AC125472	3	N	N	U	U	50
*OsHMA9*	AP008212	6	N	U	N	U	50
	Total^d^ (%)		72.2	72.2	66.7	83.3	

Note: ^a^Determined by BlastN searches performed at NCBI

^b^Changes in gene expression pattern were defined as: N = No change in gene expression; U = Significantly up-regulated expression; D = Significant down-regulated expression; and - = No expression

^C^Number of times a gene responded similarly to different heavy metal stresses; represented as percentage in the table

^d^ Number of times a heavy metal stress affected different genes in a similar fashion; represented as percentage in the table. For calculations the two copies of the *Tos17* were treated separately

In addition, among seven low-copy cellular genes (*Homeobox gene*, *DNA-binding protein*, *Elongation factor*, *HSP70*, S*NF-FZ14*, *S3*, and *YF25*), five of the genes (*Homeobox gene*, *DNA-binding protein*, *Elongation factor*, *HSP70*, and S*NF-FZ14*) showed transcriptional upregulation in all (100%) heavy metal treated plants (Fig. 1 and Table 1). Whereas, *YF25* showed significant down-regulation under Cd treatment to complete suppression under other heavy metal treatments (Cu, Cr, and Hg), and *S3* exhibited no change in expression under any of the tested heavy metal treatments.

We also tested the nine rice *HMAs* (*OsHMA1*- *OsHMA9*) and found that 7 *HMAs* showed significant up-regulation under at least one of the four heavy metal treatments (Fig. 1 and Table 1). Specifically, *OsHMA1* showed up-regulated expression in Cd and Hg-treated plants (two of the four heavy metal treatments; 50%). Similarly, *OsHMA2* showed significantly up-regulated expression in Cu-treated plants (one of the four heavy metal treatments; 25%). *OsHMA5* showed significant transcriptional activation under Cu, Cd, and Hg treatments (three of the four heavy metal treatments; 75%). *OsHMA6* and *OsHMA7* showed transcriptional activation under all four (100%) heavy metal treatments. *OsHMA8* showed significant transcriptional activation in Hg and Cr treated plants (two of the four heavy metal treatments; 50%), whereas *OsHMA9* showed significant transcriptional activation in Cd and Hg treated plants (two of the four heavy metal treatments; 50%). *OsHMA4* did not show significant transcriptional changes under any of the four heavy metal treatments, and *OsHMA3* showed no expression either in plants treated with any of the heavy metals or mock plants.

Taking the results of all four heavy metal treatments together, (i) different genes responded from none (0%) to all (100%) studied heavy metal treatments by exhibiting alterations in their respective expression patterns. Specifically, 10 of the 18 genes responded to all four heavy metal treatments by transcriptional upregulation. Interestingly, TEs and the low-copy number protein-coding genes showed more transcriptional plasticity than HMAs under heavy metal stress. (ii) With respect to the number of genes that showed transcriptional changes in response to heavy metal stress, Hg treatment induced changes in expression patterns of the maximum (83.3%) number of genes followed by Cu/Cd (72.2%), and Cr (66.7%) treatments. (iii) With respect to type (up- or down-regulation) of the gene expression changes occurring in response to the heavy metal treatment, all genes responded by up-regulation of expression, except *YF25* that showed transcriptional downregulation and *S3*, which exhibited no change in expression pattern (Table 1).

### The altered gene expression patterns were transgenerationally inherited, coupled with additional alterations in the S_1_ generation

**Fig. 2:**
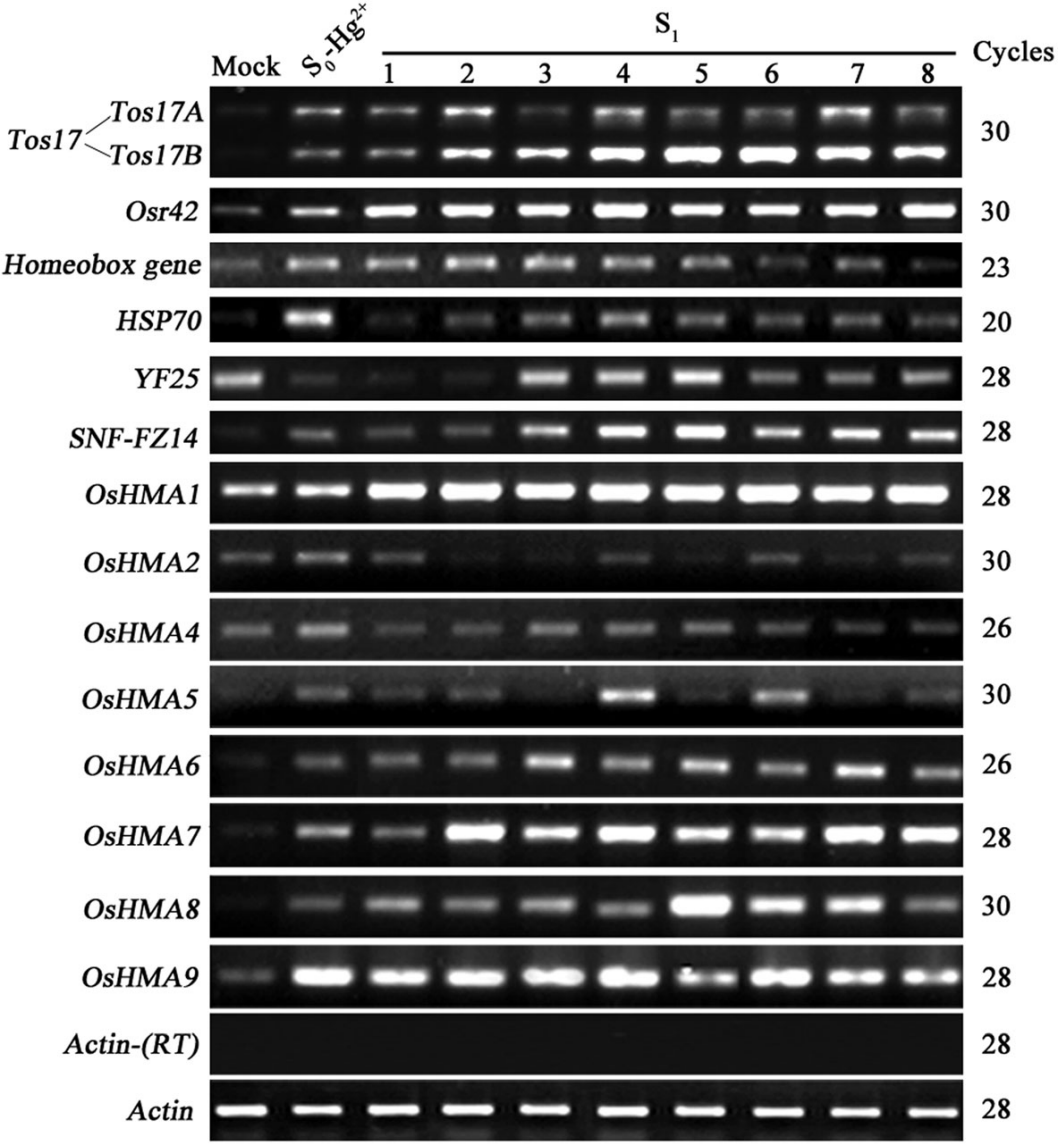
Transgenerational inheritance of altered expression states of 14 genes in a single Hg-treated S_0_ rice plant. The mock-treated plant serves as a control, and the S_0_ parental line is the reference for changes in the gene expression in response to Hg-treatment. RNA was isolated from eight S_1_ individual progeny derived from the S_0_ parent. The results were highly reproducible among the three independent RNA batches, and hence, only one was presented. Gene names are listed to the left and amplification cycles are labeled to the right of the gel. Relative band intensities were used to calculate the percent progeny falling in either of the three gene expression categories: i) inheritance of Hg-treated S_0_ pattern, ii) reversion to the mock pattern, and iii) a differential expression pattern (predominately up-regulated expression compared to the S_0_ progenitor). The rice *Actin* gene (Genbank accession # X79378) was used as a control for normalization of RNA input. Lack of genomic DNA was validated by the *Actin* gene on the template without RT

To test if the altered gene expression state of the S_0_ plants would be maintained in the next generation, we selfed a single Hg^2+^ (50 μM) treated plant, as this treatment induced gene expression changes in the majority of the studied genes (83.3%) (Table 1). Later, the leaf-tissue collected from the S_1_ seedlings growing under optimal conditions was subjected to transcript profiling of 14 genes including two transposable element genes, four cellular genes, and eight *OsHMAs*. All fourteen genes tested here showed transcriptional changes in Hg-treated S_0_ plants. We divided the expression state of S_1_ progeny into three patterns of expression: inheritance of Hg-treated S_0_ pattern, reversion to the mock pattern, and a differential expression pattern. The last category was further divided into two sub-categories: transgenerational memory (further up-regulated expression pattern) and other (cf. Fig. 2 and Table 2).

**Table 2:** Transgenerational alteration and inheritance of gene expression patterns in 8 randomly chosen S_1_ plants derived from a Hg^2+^(50 μm . L^−1^)-treated S_0_ individual

Gene name	Alteration of gene expression pattern in the S_0_ plant and its S_1_ progenies									Type and Freq. (%) of pattern^a^		
	S_0_-Hg^2+^	1	2	3	4	5	6	7	8	Inherit. of S_0_ pat.	Rev. to mock Pat.	New pat. (Trans. memory/others)
*Tos17A*	U	i	+U	i	+U	i	i	+U	i	62.5	0.0	37.5 (37.5/0.0)
*Tos17B*	U	i	+U	+U	+U	+U	+U	+U	+U	12.5	0.0	87.5 (87.5/0.0)
*Osr42*	U	+U	+U	+U	+U	+U	+U	+U	+U	0.0	0.0	100.0 (100.0/0.0)
*Homeobox gene*	U	i	i	i	i	i	r	i	r	75.0	25.0	0.0 (0.0/0.0)
*HSP70*	U	r	i	i	i	i	i	i	i	87.5	12.5	0.0 (0.0/0.0)
*YF25*	D	i	i	r	r	r	-r	-r	-r	25.0	37.5	37.5 (0.0/37.5)
*SNF-FZ14*	U	i	i	+U	+U	+U	+U	+U	+U	25.0	0.0	75.0 (75.0/0.0)
*OsHMA1*	U	+U	+U	+U	+U	+U	+U	+U	+U	0.0	0.0	100.0 (100.0/0.0)
*OsHMA2*	U	i	D	D	i	D	i	D	i	50.0	0.0	50.0 (0.0/50.0)
*OsHMA4*	U	r	r	i	i	i	r	r	r	37.5	0.0	62.5 (0.0/62.5)
*OsHMA5*	U	i	i	r	+U	i	+U	r	i	50.0	25.0	25.0 (25.0/0.0)
*OsHMA6*	U	i	i	+U	+U	+U	+U	+U	+U	25.0	0.0	75.0 (75.0/0.0)
*OsHMA7*	U	i	+U	+U	+U	+U	+U	+U	+U	12.5	0.0	87.5 (87.5/0.0)
*OsHMA8*	U	i	i	i	i	+U	+U	+U	i	62.5	0.0	37.5 (37.5/0.0)
*OsHMA9*	U	i	i	i	i	i	i	i	i	100.0	0.0	0.0 (0.0/0.0)
Average Freq. (%)^a^										41.7	6.7	41.7/10.0

Explanation of symbols: U = up-regulated gene expression in the S_0_ plant;

+U denotes further up-regulated gene expression in the S_1_ progeny plant;

D denotes down-regulated gene expression in the S_1_ progeny plants compared to the mock control

i denotes inheritance of S_0_ expression pattern in the S_1_ progeny;

r denotes reversal to Mock control expression pattern in the S_1_ progeny;

Note: ^a^ The average frequency of the specified pattern in S_1_ progeny plants. For calculations the two copies of *Tos17* were treated separately,

^a^Relative band intensities were used to calculate the percent progeny following in either of the three gene expression categories: i) inheritance of Hg- treated S_0_ pattern, ii) reversion to the mock pattern, and iii) a differential expression pattern (cf. Fig. 2)

Specifically, for the two copies of *Tos17* (*Tos17A* and *Tos17B*), the S_1_ progeny either exhibited inheritance of the S_0_ expression pattern (62.5% for *Tos17A* and 12.5% for *Tos17B*) or further up-regulation of it (37.5% for *Tos17A* and 87.5% for *Tos17B*) (Fig. 2 and Table 2). Similarly, for *Osr42*, 100% S_1_ progeny showed further up-regulation of the S_0_ expression pattern.

Out of four low-copy number protein-coding genes (Fig. 2 and Table 2), for *Homeobox* gene and *HSP70*, the majority of S_1_ progeny (75% for *Homeobox* gene and 87.5% for *HSP70*) exhibited stable inheritance of the S_0_ expression pattern, and the remainder (25% for *Homeobox* gene and 12.5% for *HSP70*) showed reversal to the mock expression pattern. On the other hand, *YF25* which showed significant down-regulation in the S_0_ generation_,_ exhibited inheritance of the altered expression state, reversal and novel gene expression pattern in the S_1_ progeny at frequencies of 25, 37.5, and 37.5%, respectively. For *SNF-FZ14,* which showed transcriptional activation in S_0_ generation exhibited further up-regulated expression pattern in the majority (75%) of the S_1_ plants and exhibited inheritance of the altered expression state in the remaining 25% of the progeny.

For the eight *OsHMAs* tested (Fig. 2 and Table 2), all showed up-regulated expression in S_0_ plants compared to the mock-treated plants, but differences were found in the S_1_ generation: *OsHMA1* showed further up-regulated expression in 100% progeny. *OsHMA2* showed 50% inheritance of up-regulated expression and reversal to the basal expression state in 50% of the progeny. *OsHMA4* showed the inheritance of the S_0_ expression state in 37.5% of the progeny and reversal to the basal expression state in 62.5% of the progeny. *OsHMA5* showed inheritance, reversal and further up-regulated expression patterns in 50, 25, and 25% of the S_1_ plants, respectively; *OsHMA6*, *OsHMA7*, and *OsHMA8* showed inheritance of the altered expression state in 25, 12.5, and 62.5% of the S_1_ progeny, and further up-regulated expression in 75, 87.5, and 37.5% of the progeny. *OsHMA9* showed significantly up-regulated expression in the S_0_ plants, and all S_1_ progeny (100%) inherited the expression pattern.

In summary, we found that for those genes that showed changes in expression in the S_0_, two major gene expression patterns were manifest in the S_1_ progeny: either inheritance of the S_0_ expression pattern (41.7%) or adaptation to a new expression pattern (51.7%). However, the maintenance of change in gene expression varied among the genes tested. For instance, some genes (*Tos17A*, *Homeobox gene*, *HSP70*, *OsHMA2*, *OsHMA5*, *OsHMA8* and *OsHMA9*) exhibited inheritance of the expressed state from S_0_ to S_1_ generations in ≥50% progeny plants, whereas other genes (*Tos17B*, *Osr42*, *SNF-FZ14*, *OsHMA1*, *OsHMA2*, *OsHMA6*, and *OsHMA7*) showed a further up-regulated expression in ≥50% progeny plants suggesting genetic memory of the altered expression pattern gained in response to the heavy metal treatment that is transmitted to the next generation.

### The altered gene expression states were transgenerationally persistent, coupled with the genetic memory in the S_2_ generation

**Fig. 3:**
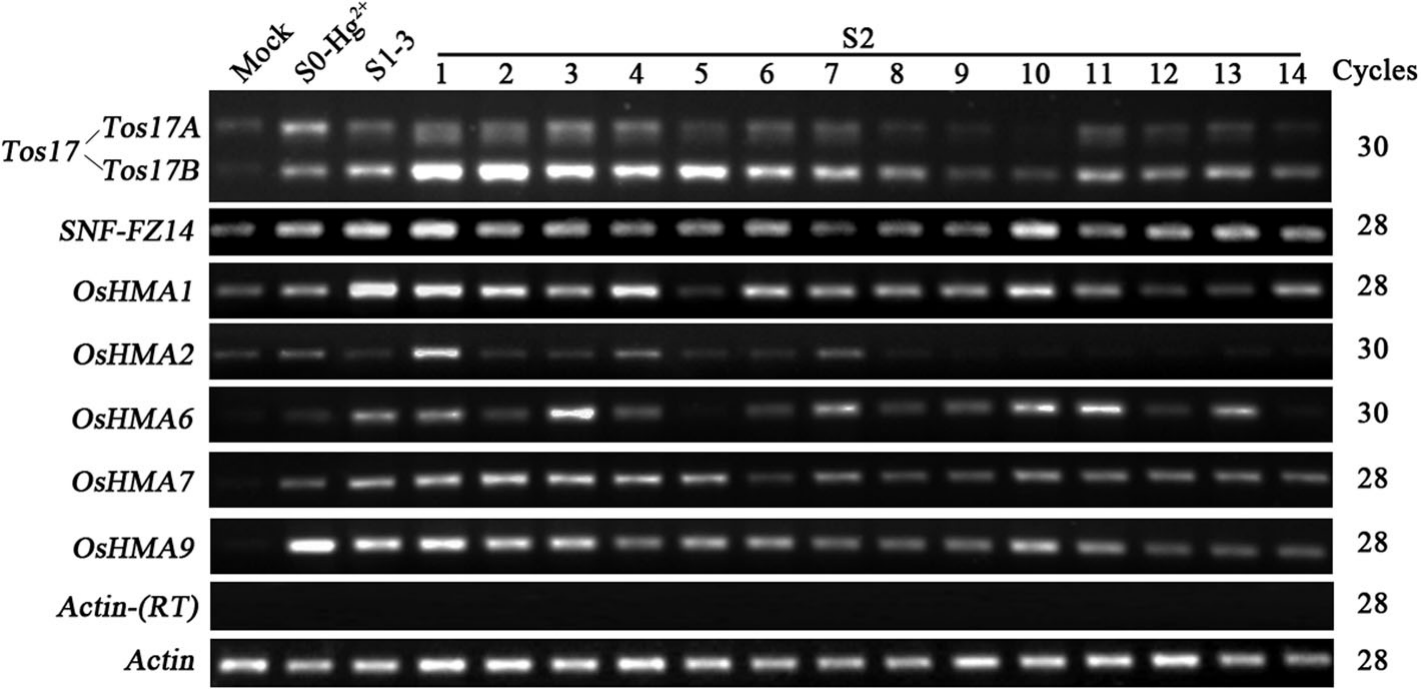
Transgenerational inheritance of altered expression states of seven genes in a single S_1_ rice plant. As evidence of inheritance of the expression states, the S_0_ and S_1_ plants are used as a reference as well as the mock control (no metal treatment). A total of 14 S_2_ individuals were examined to determine the expression of the *Tos17A*, *Tos17B*, *SNF-FZ14* and five *OsHMA* transporters in the second generation. Gene names are listed to the left and amplification cycles are labeled to the right of the gel. Relative band intensities were used to calculate the percent progeny falling in either of the four gene expression categories: i) inheritance of the S_1_ expression state, ii) reversion to the S_0_ expression state, iii) reversion to the mock expression state, and iv) a novel expression pattern. The rice *Actin* gene (Genbank accession # X79378) was used as a control for normalization of RNA input. Lack of genomic DNA was validated by the *Actin* gene on the template without RT

To further test if the altered expression states are transgenerationally persistent, we selected one S_1_ plant (plant #3) that exhibited all three expression patterns for several of the tested genes, i.e., inheritance of the S_0_ expression pattern, reversal to the basal expression pattern and adaption of a new expression pattern, to obtain S_2_ progeny. To study the expression pattern, we performed the RT-PCR analysis of seven genes (*Tos17*, *SNF-FZ14*, *OsHMA1*, *OsHMA2*, *OsHMA6*, *OsHMA7*, and *OsHMA9*) in the leaf-tissue of 14 randomly selected S_2_ individuals grown under optimal conditions. The seven genes selected for RT-PCR analysis showed increased expression in the S_0_ generation and exhibited different expression patterns in the S_1_ generation. Of the seven genes tested, we identified four gene expression patterns in the S_2_ progeny, i.e., the inheritance of the S_1_ expression state, reversion to the S_0_ expression state, reversion to the mock expression state, and a novel expression pattern (Fig. 3 and Table 3). We observed the majority of S_2_ progeny inherited the expression state of the S_1_ progenitor, 36.6% progeny showed inheritance of the S_1_ expression state, 22.3% progeny reverted to the S_0_ expression state, 22.3% progeny showed reversal to the basal expression state (similar to mock), and the remaining 18.8% progeny adopted a new expression pattern.

**Table 3:** Transgenerational alteration and inheritance of gene expression patterns in the 14 randomly chosen S_2_ plants derived from one S_1_ individual #3 (S_1_–3), which was derived from a single Hg^2+^ (50 μm)-treated S_0_ individual

Gene name			Gene expression patterns in the S_2_ progeny														Frequency (%)^b^			
	S_0_-Hg^2+^	S_1_–3	1	2	3	4	5	6	7	8	9	10	11	12	13	14	Inherit. of S_1_ pat.	Rev. to S_0_ pat.	Rev. to mock pat.	New pat. (Trans. Memory/others)
*Tos17A*	U	U	U	U	U	U	r	U	U	r	r	r	U	r	U	r	57.1	0.0	42.9	0.0 (0.0/0.0/)
*Tos17B*	U	+U	++U	++U	++U	++U	++U	++U	++U	+U	U	U	+U	+U	+U	+U	35.7	0.0	14.3	50.0 (50.0/0.0)
*SNF-FZ14*	U	+U	++U	U	U	U	U	U	r	r	r	++U	U	U	U	U	0.0	21.4	64.3	14.3 (14.3/0.0)
*OsHMA1*	U	+U	+U	+U	U	+U	r	U	U	U	U	+U	U	r	r	U	28.6	50.0	21.4	0.0 (0.0/0.0)
*OsHMA2*	U	D	+U	r	r	U	r	r	U	-D	-D	-D	-D	-D	-D	-D	0.0	14.3	28.6	57.1 (0.0/57.1)
*OsHMA6*	U	+U	+U	U	++U	U	++U	U	+U	U	U	++U	++U	U	+U	r	21.4	42.9	7.1	28.6 (28.6/0.0)
*OsHMA7*	U	+U	+U	+U	+U	+U	+U	U	+U	U	U	+U	U	U	U	U	50.0	50.0	0.0	0.0 (0.0/0.0)
*OsHMA9*	U	U	U	U	U	U	U	U	U	U	U	U	U	U	U	U	100.0	0.0	0.0	0.0 (0.0/0.0)
Average Freq. (%)^a^																	36.6	22.3	22.3	18.8 (11.6/7.2)

Explanation of symbols: U denotes up-regulated gene expression in the S_0_ plant, and kept the trend in S_1_–3 and S_2_ individuals;

+U denotes further up-regulated gene expression in the S_1_–3 and kept the trend in S_2_ individuals;

++U denotes further up-regulated gene expression in S_2_ individuals compared to their S_1_ progenitor;

D =denotes down-regulated gene expression in the S_1_–3 compared to the Mock control;

-D denotes further down-regulated gene expression in S_2_ individuals compared to their S_1_ progenitor;

r denotes reversal to Mock control expression pattern in the S_2_ progeny;

Note: ^a^The average frequency of the specified pattern in S_2_ progeny plants. For calculations the two copies of *Tos17* were treated separately,

^b^Relative band intensities were used to calculate the percent progeny following in either of the four gene expression categories (cf. Fig. 3)

On gene by gene basis, the proportions of S_2_ progeny following one of the four expression patterns (see above) also varied, for instance, in case of *Tos17A*, *OsHMA7*, and *OsHMA9*, ≥50% S_2_ progeny exhibited inheritance of the S_1_ expressed state. For *OsHMA1* and *OsHMA7*, ≥50% S_2_ progeny showed reversal to the expression state of the S_0_ progenitor. Similarly, for *SNF-FZ14* 64.3% S_2_ progeny showed a reversal to the basal expression state. Whereas, in the case of *Tos17B* and *OsHMA6* respectively 50 and 28.6% S_2_ progeny showed a further up-regulation of the S_1_ expression pattern.

Collectively, these results suggested that the altered gene expression states induced by heavy metal stress are heritable (11.6%; Table 3), and hence indicates transgenerational memory is involved. Additionally, the progeny also appears to maintain the upward trend of induced expression in response to heavy metal stress.

### DNA methylation changes of *Tos17* and its transgenerational effect

**Fig. 4:**
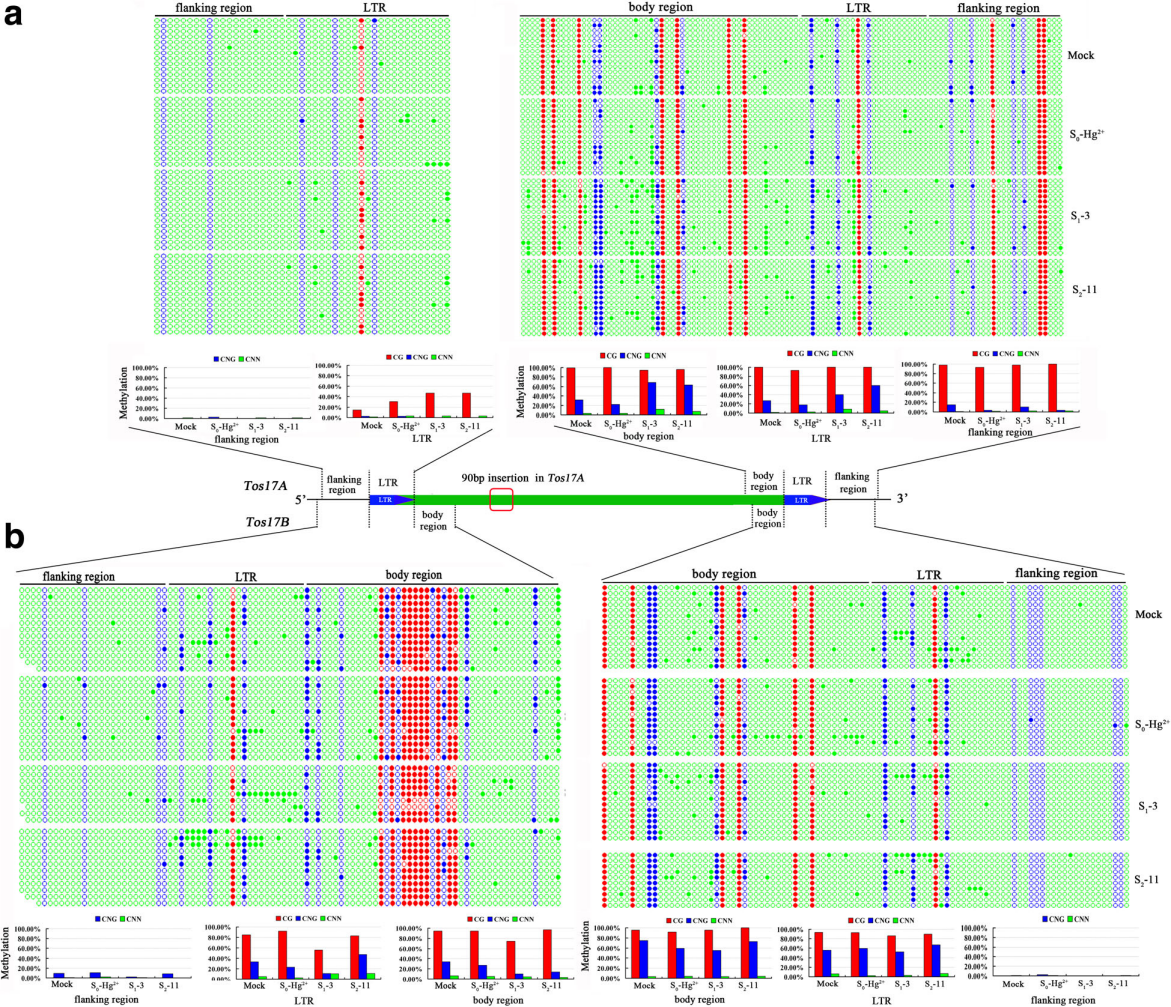
DNA methylation status of the *Tos17A* (**a**) and *Tos17B* (**b**) determined by bisulfite sequencing, respectively, in mock and the Hg-treated S_0_ plant, and its two successive offspring: S_1_–3 (S_1_ generation plant # 3) and S_2_–11 (S_2_ generation plant # 11). Specific primers were used on the bisulfite-treated rice genomic DNA to amplify six sites from the two *Tos17* (Transposon of *Oryza sativa* 17) copies in the rice genome (cf. Additional file 2: Table S2). Each copy of *Tos17* was amplified from six genomic sites: 3 from the 5′-LTR region (i.e., flanking region, LTR, and body region, expect the body region of *Tos17A*) and 3 from the 3′-LTR region (i.e., flanking region, LTR, and body region). Subsequently, 10 to 15 clones for each PCR product were sequence analyzed, and the methylation levels per site for each of the three cytosine contexts (CG, CHG, and CHH) were calculated and expressed as a percentage (%). Methylation level was calculated by dividing the number of non-converted (methylated) cytosines with the total number of cytosines underlying a sequenced region. In the picture, each DNA sequences was represented by a string of dots, where filled dots represent methylated cytosines and the open dots represent unmethylated cytosines

To further explore whether DNA methylation was also altered due to heavy metal stress and to explain its inheritance across generations, we chose Hg-treated S_0_ plants, one S_1_ individual (#3) and one S_2_ individual (#11) to investigate the methylation state and its transmission. We chose *Tos17* as a representative gene to test because both copies of *Tos17* showed induced expression in the S_0_ and the progeny kept the trend through two successive generations. We analyzed cytosine methylation patterns of *Tos17A* and *Tos17B* by bisulfite sequencing (Fig. 4). Specifically, we inspected the 5′- LTR and its immediate upstream and downstream regions as well as the 3′-LTR and its immediate upstream and downstream regions for *Tos17A* and *Tos17B* located on chromosomes 7 and 10, respectively. The results of bisulfite sequencing are presented in Fig. 4, and some salient observations are described: (i) The region immediately upstream of 5′-LTR in *Tos17A* showed no change in DNA methylation in the S_0_ plants and the S_1_/S_2_ progeny; the LTR region was slightly methylated at CG and CNG regions in the mock-treated plants and showed CG hypermethylation in S_0_ plants, further hypermethylation in S_1_ progeny and inheritance of methylation state in S_2_ plants. (ii) The 3′-LTR and its flanking regions in *Tos17A* showed CG hypermethylation and partial methylation for CNG and CNN sequences in the mock plants. However, the CG methylation pattern remained unchanged in the S_0_, S_1_, and S_2_ plants. A slight loss of CNG methylation was observed in the body and LTR regions in S_0_ plants, but increased methylation levels were observed in the S_1_ progeny. In the S_2_ progeny, a slight decrease in methylation pattern in the body region and hypermethylation in the LTR region was observed (Fig. 4a). (iii) The flanking region upstream of the 5′-LTR of *Tos17B* was unmethylated in the mock plants and showed slight de novo methylation in CNG sequences in the S_0_ plants, a pattern which disappeared in the S_1_ progeny. In contrast, the 5′-LTR and the downstream body regions of *Tos17B* showed heavy methylation in CG sequences, and slight to moderate increases in CNN and CNG methylation compared to the mock control. A decrease of CG methylation was observed in the S_1_, as well as a decrease in CNG methylation in both S_0_ and S_1_, but an increase in CNG methylation was found in the S_2_ progeny (Fig. 4b). Taken together, the results of bisulfite sequencing at *Tos17A* and *Tos17B* confirmed that DNA methylation changes occur in response to the heavy metal treatment and also showed transgenerational inheritance. Furthermore, the major pattern of DNA methylation changes is CNG hypomethylation in the S_0_, which showed different transgenerational effects in either the 3′-region of *Tos17A* or 5′-region of *Tos17B*.

**Fig. 5:**
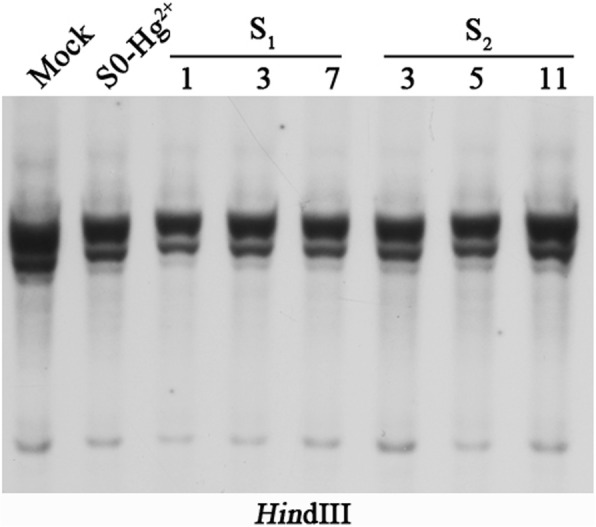
Determination of the *Tos17* copy number using gel-blot analysis in a Hg-treated S_0_ plant and its two successive offspring S_1_ (1, 3, and 7) and S_2_ (3, 5, and 11). The results showed that *Tos17* stayed inactive, which is evident from the consistent copy number maintained in individuals of the S_0_, S_1_, and S_2_ generations observed using a single LTR retrotransposon *Tos17* specific probe (cf. Additional file 1: Table S1)

The gene expression and DNA methylation of two copies of *Tos17* changed under heavy metal stress and showed transgenerational memory of the stress. In addition, under certain circumstances, some of the epigenetically silenced TEs are known to become activated and then transpose. TE activity is often causally linked to the compromised repressive epigenetic state in which cytosine DNA methylation is a critical component. We, therefore, analyzed *Tos17* mobility in the S_0_, S_1_, and S_2_ generations by Southern blotting. The results showed that *Tos17* stayed inactive, which is evident from the consistent copy number maintained in individuals from the S_0_, S_1_, and S_2_ generations (Fig. 5).

## Discussion and conclusions

In this study, locus-specific gene expression changes and the transgenerational effect of heavy metal stress in rice were analyzed. For this purpose, we chose two retrotransposons, seven protein-coding genes, and nine rice *OsHMAs*, most of them except seven *OsHMAs* were analyzed in a previous study of the transgenerational inheritance of modified DNA methylation patterns in response to heavy metal stress [41]. In the present study, we addressed whether the altered expression state of the target genes in response to heavy metal stress is transgenerationally inherited and whether different kinds of genes have common or specific responses to the same heavy metal stress. Based on our previous findings, we chose a single dose of each heavy metal that induced maximum DNA methylation changes [41], and also included a lower dose of heavy metal to study its effect on the transcription and methylation states. The results showed that 16 of 18 genes exhibited up-regulated expression upon treatment with at least one heavy metal (Fig. 1), indicating that a common response might exist for most genes upon heavy metal stress. Our previous data showed that *Tos17* and *Osr42* exhibit up-regulated expression in response to nitric oxide (NO) treatment in rice [43]. It indicates that these two retrotransposons exhibit transcriptional plasticity to cope with stress. For *Tos17*, we examined the transcriptional response of the two genomic copies, and both of them showed activation in response to the heavy metal treatment (Fig. 1). It is the first time that the transcriptional activation of both copies of *Tos17* was demonstrated in response to abiotic stress.

All *OsHMAs* except *OsHMA3* showed significantly up-regulated expression in response to at least one kind of heavy metal treatment, which suggested that these might all be involved in the heavy metal transport. Previous reports suggested *OsHMA1* to be exclusively involve in Zn transport [26], however, in the present study, it showed significantly up-regulated expression in Hg treated rice plants, implicating that it might be also involved in transporting Hg. Similarly, *OsHMA2* was formerly reported to be expressed in the root maturation zone and to function in the root-shoot translocation of Zn and Cadmium (Cd) [28, 44]. In the present study, *OsHMA2* showed transcriptional activation in Cu treated rice plants, suggesting its potential role in copper (Cu) transport. *OsHMA3* was localized to tonoplast in the root cells and was found to be responsible for Cd sequestration in vacuoles [29, 30, 45]. In the present study, *OsHMA3* showed no expression in rice shoots or induction after Cu, Cd, Cr or Hg treatment, which is consistent with a recent report that it was not induced in roots and shoots of Cr-treated rice plants [46]. However, overexpression of *OsHMA3* was shown to enhance Cd tolerance in rice [47], and a loss-of-function allele was shown to accumulate Cd in grains and shoots [48]. Interestingly, it was recently shown that *OsHMA3* driven under the control of the *OsHMA2* promoter was successful at reducing Cd accumulation in rice grains [28]. *OsHMA4* is localized to the vacuolar membrane, and its expression was shown to be induced by long-term Cu treatment and suppressed by Cu deficiency [31] suggesting its role in Cu sequestration in vacuoles and consequently Cu tolerance. In the present study, *OsHMA4* was only slightly induced by Cu treatment, which is in conformity with the previous reports where *OsHMA4* was only shown to be induced by long-term Cu treatment [27, 31]. *OsHMA5* was mainly expressed in the roots at the vegetative stage, and its expression was shown to be up-regulated by the excess of Cu and other metals such as Zn, Fe, and Mn [32]. Here, we report that *OsHMA5* is not expressed in the shoots of mock-treated plants, but is induced in the presence of Cu, which is consistent with a previous study [32]. Additionally, we noticed that *OsHMA5* exhibits induced expression in the presence of Cd and Hg as well. There are few reports on the function of *OsHMA6*, *OsHMA7*, and *OsHMA8*. These genes are largely silent in the shoots and only exhibited transcriptional activation under heavy metal stress. Although detailed functions are not known for these genes, our data suggest they may also play a role in heavy metal detoxification. Previous reports showed that *OsHMA9* is mainly expressed in vascular tissues and its expression could be induced by high concentrations of Cu, Zn or Cd [27]. In the present study, *OsHMA9* showed significant transcriptional activation in Cd and Hg treated plants, and a slight up-regulation in Cu treated plants. Our data support an additional role for *OsHMA9* in Hg efflux.

To confirm and extend our findings, we tested whether the altered gene expression state of S_0_ plants was transgenerationally inherited by the S_1_ and S_2_ progeny. We reported an average inheritance rate of 41.7% in the S_1_ and 36.6% in the S_2_ (Figs. 2, 3 and Tables 2, 3). However, the rate of inheritance varied depending on gene in question. A majority of the genes tested showed up-regulated expression in the S_1_ (41.7%) and about 11.6% maintained the trend of up-regulated expression and exhibited further up-regulation in the S_2_. It indicates that the progeny maintained a memory of the altered expression state of the progenitors even after removal of the heavy metal. Recently, some studies showed a clear connection between the ethylene signaling and response to heavy metal stress in diverse plant species [49–51]. We have not evaluated this aspect in the present study, but believe it is worthy of checking the transcriptional pattern of ethylene biosynthesis and signaling genes in heavy metal treated plants and study the transgenerational inheritance of the expression pattern.

The traditional concept of epigenetics refers to heritable changes in gene expression without an accompanying change in the DNA sequence. Recent research advocates inclusion of the ‘memory concept’ in the formal definition of epigenetics, as even after the disappearance of the initial stress signal, the DNA and/or chromatin modifications are transmitted to maintain the altered transcriptional state from one generation to another [52, 53]. Several studies showed that epigenome is remodeled in plants upon exposure to diverse stresses and DNA methylation pattern is most likely to respond [54–59]. It has been proposed that the DNA methylation state is only partially transmitted to the immediate offspring, as part of it resets during sexual reproduction, which in turn limits the transmission of the acquired epigenetic alterations from parents to offspring [60, 61]. However, our previous research demonstrated that the heavy metal-induced DNA methylation changes in rice are inheritable for at least two successive generations [41]. Here, we monitored the DNA methylation changes under heavy-metal stress in two copies of *Tos17* and studied the transgenerational inheritance of epigenetic changes by bisulfite sequencing (Fig. 4). We observed that the major DNA methylation change in *Tos17* is CNG hypomethylation, which showed variable inheritance patterns in the 3′- and 5′-regions of the two genomic copies of *Tos17* (*Tos17A* and *Tos17B*). These observations conform with our previous findings where CNG hypomethylation was most prevalent in response to heavy metal stress and showed at least partial inheritance of the epigenetic changes [41, 43]. DNA methylation changes are associated with changes in gene expression. For instance, *A. thaliana* mutants defective in DNA methylation showed that regulation of phosphate-starvation-responsive genes requires changes in the DNA methylation pattern [59]. Thus, we set out to find the relationship between DNA methylation and gene expression. Our data suggest that there is no direct correlation between the methylation status and gene expression for *Tos17*. Moreover, *Tos17* stayed silent over three generations, which indicates that the methylation changes in *Tos17* are not sufficient for its activation followed by transposition. However, it is unclear whether the heritable change in gene expression is related to methylation changes as there can be locus-specific changes in methylation. Moreover, our study was limited to *Tos17A* and *Tos17B*.

Interestingly, recent research has proposed a key role for dynamic changes in chromatin substructure in transgenerational memory of gene expression changes in response to various stresses [62–64]. In line with this research, maize-researchers showed that stress-induced changes in chromatin structure activate transposable elements, and new transposition events contribute to altered phenotypes observed in the progeny [65]. Several studies indicated that DNA methylation and small interfering (si) RNAs might play a role in transgenerational epigenetic memory, i.e., modification in gene expression patterns that are transmittable across generations via the germline [37, 66–69]. Therefore, we expect a role for siRNA in the observed transgenerational memory of heavy-metal induced transcriptional and epigenetic changes in the rice genome. However, as noted by Probst and Mittelsten [63], while the concept of transgenerational memory is attractive, it is difficult to determine the actual mechanism contributing to it and the number of generations in which it persists.

## Methods

### Plant material

*O. sativa* L. ssp. *japonica*, cv. Matsumae, a cultivated rice, used in the present study was initially obtained from Japan and has since been propagated for more than twenty generations in our laboratory. For the experiments elaborated here, seeds were thoroughly washed with distilled water and germinated in the dark at 28 °C in Petri dishes containing distilled water. After two days incubation, seedlings were transferred to a greenhouse maintained at 26 °C under a 12 h photoperiod.

### Heavy metal treatment

The ten-day-old, seedlings were subjected to different heavy metal treatments: Cu^2+^ (50 μM or 1000 μM CuSO_4_), Cd^2+^ (50 μM or 1000 μM CdCl_2_), Cr^3+^ (50 μM or 1000 μM CrCl_3_) or Hg^2+^ (50 μM or 1000 μM HgCl_2_) in Hoagland nutrient solution for a week. As several microelements in Hoagland nutrient solution are either used as sulfates or chlorides, and the pH of the solution is also adjusted using sulfuric acid, so we made no attempts to balance the sulfate and chloride ions in the Hoagland solution. Additionally, the treatments are similar to the one reported in our previous work [41]. Mock controls were grown in parallel in the Hoagland nutrient solution. After treatment, seedlings were transplanted to the field. Leaf samples were harvested at different time points in liquid nitrogen and stored at − 80 °C until used. The plants were marked “stressed S0”. Panicles of several selected stressed and mock plants were bagged for self-pollination and seeds were collected to produce the next generation of plants, which were labeled as S1. In a similar way, S2 generation plants were produced, and the seeds were harvested.

### Reverse-transcription PCR (RT-PCR) analysis

RT-PCR was performed essentially as reported in Liu et al. [70]. In brief, total RNA was isolated from expanded young leaves using Trizol reagent (Invitrogen) following the manufacturer’s instructions. RNA was converted to cDNA using Super ScriptTM RNase H reverse transcriptase kit (Invitrogen), and subjected to RT-PCR analysis using gene-specific primers (Additional file 1: Table S1). The rice *Actin* gene (Genbank accession # X79378) was used as the control for normalization of RNA input. Gene-specific primers were designed using Primer 3 (http://bioinfo.ut.ee/primer3/) and are listed in Additional file 1: Table S1. Different cycle numbers were used for different genes to ensure amplifications stay within the linear range for each gene. For S_0_ samples, we pooled seedings and used three technical replications to check the gene expression changes. Whereas, for the S_1_ and S_2_ individuals, three batches of independently prepared total RNAs were used as technical replications. The amplified products were visualized via agarose gel electrophoresis and ethidium bromide staining.

### Bisulfite sequencing of the *Tos17* loci

Genomic DNA was extracted from fully expended rice leaves and was given a bisulfite treatment [71]. Briefly, an EZ DNA Methylation-Gold Kit from Zymo Research was used to treat 5 μg of genomic DNA. The PCR primers, which were used to amplify bisulfite-converted genomic DNA for the two copies of the *Tos17* (Transposon of *Oryza sativa* 17), are listed in Additional file 2: Table S2. From 10 to 15 clones for each sample were sequence analyzed. The methylation level was expressed as the percentage (%) per site for each of the three cytosine contexts (CG, CHG, and CHH). Methylation level was calculated by dividing the number of non-converted (methylated) cytosines with the total number of cytosines underlying a sequenced region. The sequences were analyzed by the Kismeth program (http://katahdin.mssm.edu/kismeth/revpage.pl), and the results were presented as histograms.

### Southern blotting

Genomic DNA was isolated from fully expanded leaves of heavy metal-stressed and mock control rice plants by a modified CTAB method [72] and purified by phenol extraction. For the transposon activity analysis, 5 μg of genomic DNA was digested with *Hin*d III (NEB) and resolved on 1% agarose gel. Subsequently, DNA was transferred to Hybond N+ nylon membranes (Amersham Pharmacia Biotech, Piscataway, New Jersey) via alkaline transfer, as recommended by the manufacturer. Only one *Tos17* copy was used as a probe in the present study (see Additional file 1: Table S1). For probe preparation, the Tos17 fragments were amplified via PCR at annealing temperature 59 °C. The authenticity of the PCR products was confirmed by DNA sequencing. The fragments were gel-purified and labeled with fluorescein-11-dUTP using the Gene Images random prime-labeling module from Amersham Pharmacia Biotech. Hybridization signal was detected by the Gene Images CD^2+^P-Star detection module (Amersham Pharmacia Biotech) after two stringent washes with 0.2 × SSC and 0.1% SDS for 50 min each. Subsequently, the membrane was exposed to X-ray film.

## Additional files

Additional file 1: **Table S1.** List of gene-specific primers used for RT-PCR analysis and amplification of probe used for Southern blotting. (DOC 50 kb)

Additional file 2: **Table S2.** List of primers used for bisulfite sequencing of *Tos17*. (DOC 39 kb)

## Data Availability

All data generated or analyzed during this study are included in this published article [and its supplementary information files].

## Abbreviations

Cd: Cadmium
Co: Cobalt
Cr: Chromium
CTAB: Cetyltrimethylammonium bromide
Cu: Copper
Hg: Mercury
HMA: Heavy Metal-transporting P-type ATPases
NO: Nitric oxide
Pb: Lead
RT-PCR: Reverse transcription-polymerase chain reaction
SDS: Sodium dodecyl sulfate
SSC: Saline sodium citrate
TE: Transposable element
Zn: Zinc
