# Supplementary material for: Transgenerational memory of gene expression changes induced by heavy metal stress in rice (Oryza sativa L.)
Source: BMC Plant Biol. 2019 Jun 27;19:282. doi: 10.1186/s12870-019-1887-7 (PMC6598230; doi:10.1186/s12870-019-1887-7)
Supplement: Supplementary file 1 — Table S1. List of gene-specific primers used for RT-PCR analysis and amplification of probe used for Southern blotting. (DOC 50 kb) [file 12870_2019_1887_MOESM1_ESM.doc]

Additional file 1

**Transgenerational Memory of Gene Expression Changes Induced by Heavy Metal Stress in Rice (*Oryza sativa* L.)**

Weixuan Cong^1†^, Lei Xu^1†^, Yiling Miao^1†^, Yunhong Zhang^1^, Chunlei Yuan^1^, Junmeng Wang^1^, Tingting Zhuang^1^, Xiuyun Lin^2^, Lili Jiang^1^, Ningning Wang^3^, Jian Ma^3^, Karen A. Sanguinet^4^, Bao Liu^1^, Sachin Rustgi^4,5*^, Xiufang Ou^1*^

^1^ Key Laboratory of Molecular Epigenetics of MOE and Institute of Genetics & Cytology, Northeast Normal University, Changchun 130024, China.

^2^ Jilin Academy of Agricultural Sciences, Changchun 130033, China.

^3^Jilin Agriculture University, Changchun 130000, China.

^4^Department of Crop and Soil Sciences, Washington State University, Pullman, WA 99164, USA.

^5^Clemson University Pee Dee Research and Education Center, 2200 Pocket Road, Florence SC 29506, USA.

^†^These authors contributed equally to this work.

*Correspondence:

XFO: e-mail: [ouxf074@nenu.edu.cn](mailto:ouxf074@nenu.edu.cn); fax: +86-431-85099822

SR: e-mail: [srustgi@clemson.edu](mailto:srustgi@clemson.edu); fax: +1-843-662-2112

**Table S1. List of gene-specific primers used for RT-PCR analysis.**

| Gene name | Genbank accession | Forward primer (5’-3’) | Reverse primer (5’-3’) | Annealing  temperature(°C) |
| --- | --- | --- | --- | --- |
| *OsActin* | X79378 | cgtgtgcgataatggaactg | tctgggtcatcttctcacga | 55 |
| *Tos17** | AC087545 (*Tos17A*)  AP008213 (*Tos17B*) | aaagggaaactcagcgaaca | gagggcacatagtggagagc | 60 |
| *Osr42* | AF458768 | ccacagatcatcatttctgacc | ccccttgaagactgacttgc | 58 |
| *Homebox gene* | AB007627 | ttgatggaaatgatgggtca | actgcatcgtgcatcaaaac | 55 |
| *DNA-binding protein* | X88798 | agaatgccactcctcctgtg | gtcctcccttctgtgctgag | 60 |
| *Elongation factor* | D12821 | acctctccggcaagacctac | ttacaagccgctctgcagtt | 60 |
| *HSP70* | X67711 | cccatcttggtggtgaagat | gtcctcagcagacacgttca | 58 |
| *SNF-FZ14* | DQ239432 | tgaggctgtcagcatgatct | atctgaggatgttgcgcttc | 56 |
| *S3* | AY328087 | ggtgcactgcttcttcattg | tcgagttgtcctcgtcctct | 58 |
| *YF25* | DQ239435 | ccggatggaagaagagatca | tggagctcaaggcgatattt | 55 |
| *OsHMA1* | AP003935 | gaaagtgaagctctagctgttgc | cttgattcatgatctcccgtaag | 58 |
| *OsHMA2* | AP004278 | accagctgattacaaacaagcat | cagtcctttacttcctcgacctc | 60 |
| *OsHMA3* | AP005246 | CTTCTTTACTGGATTGCAAGCAT | GGTCAGCATAACCGACTTGATG | 58 |
| *OsHMA4* | AP004184 | tgctggggaaatatctggag | cttctgaactggagcccttg | 58 |
| *OsHMA5* | AL606647 | cggtgaatgagaatggtgtg | tcttgtcgaaaacgatgcag | 55 |
| *OsHMA6* | AP004836 | ggggctaatcatggggtact | gaactcctgtcttcgcgttc | 58 |
| *OsHMA7* | AP004376 | ccaaccttcgagactcttca | gcatgggacctcagtaaatg | 55 |
| *OsHMA8* | AC125472 | ataatggaggaggccgaact | cctgccttttcatgtggagt | 55 |
| *OsHMA9* | AP008212 | gacaaaacaggcacactaacaca | cattaaacccacaaaatcatcgt | 60 |

***This primer pair was used for both RT-PCR and amplification of probe for gel-blot analysis.
