# Supplementary material for: Transgenerational memory of gene expression changes induced by heavy metal stress in rice (Oryza sativa L.)
Source: BMC Plant Biol. 2019 Jun 27;19:282. doi: 10.1186/s12870-019-1887-7 (PMC6598230; doi:10.1186/s12870-019-1887-7)
Supplement: Supplementary file 2 — Table S2. List of primers used for bisulfite sequencing of Tos17. (DOC 39 kb) [file 12870_2019_1887_MOESM2_ESM.doc]

Additional file 2

**Transgenerational Memory of Gene Expression Changes Induced by Heavy Metal Stress in Rice (*Oryza sativa* L.)**

Weixuan Cong^1†^, Lei Xu^1†^, Yiling Miao^1†^, Yunhong Zhang^1^, Chunlei Yuan^1^, Junmeng Wang^1^, Tingting Zhuang^1^, Xiuyun Lin^2^, Lili Jiang^1^, Ningning Wang^3^, Jian Ma^3^, Karen A. Sanguinet^4^, Bao Liu^1^, Sachin Rustgi^4,5*^, Xiufang Ou^1*^

^1^ Key Laboratory of Molecular Epigenetics of MOE and Institute of Genetics & Cytology, Northeast Normal University, Changchun 130024, China.

^2^ Jilin Academy of Agricultural Sciences, Changchun 130033, China.

^3^Jilin Agriculture University, Changchun 130000, China.

^4^Department of Crop and Soil Sciences, Washington State University, Pullman, WA 99164, USA.

^5^Clemson University Pee Dee Research and Education Center, 2200 Pocket Road, Florence SC 29506, USA.

^†^These authors contributed equally to this work.

*Correspondence:

XFO: e-mail: [ouxf074@nenu.edu.cn](mailto:ouxf074@nenu.edu.cn); fax: +86-431-85099822

SR: e-mail: [srustgi@clemson.edu](mailto:srustgi@clemson.edu); fax: +1-843-662-2112

**Table S2.** List of primers used for bisulfite sequencing of *Tos17*.*

| Primer name | Primer sequence | Product size（bp） |
| --- | --- | --- |
| *Tos17A*-5-F | TGGAYAGATYAAGYYTAAYTTGGGAAG | 266 |
| *Tos17A*-5-R | RACCATTRCTCTRATACCATCTTAACT |
| *Tos17A*-3-F | YGGAGYTATAYAAATYGYYAATGAT | 497 |
| *Tos17A*-3-R | RCAATCRARTAAAAAAACATRCACCT |
| *Tos17B*-5-F | YTYGGATGTYTTTAGATGTAYTTAAAAAAGG | 443 |
| *Tos17B*-5-R | TARCCCACRARRCRACRRTRAAAARRACA |
| *Tos17B*-3-F | ATGATYYAATYAAGYATGAATTGAYGAAGYATATTG | 461 |
| *Tos17B*-3-R | AAARTTCARATRCTTRTTCACRTCTTCATRCAT |

**Tos17* has two copies, *Tos17A* and *Tos17B*, the former is located on chromosome 10 between 80,982 bp to 85,185 bp on BAC AC087545 (it possesses a 90 bp insertion) and the latter is located on chromosome 7 between 26,641,215 bp to 26,645,328 bp on BAC AP008213 (cf. Ding et al. 2007).

Ding Y, Wang X, Su L et al (2007) SDG714, a Histone H3K9 Methyltransferase, Is Involved in Tos17 DNA Methylation and Transposition in Rice. Plant Cell 19:9-22.
